# Site-specific antitumour effects of 2 pyrimidinone compounds in rats.

**DOI:** 10.1038/bjc.1986.182

**Published:** 1986-08

**Authors:** A. M. Eggermont, R. L. Marquet, R. W. De Bruin, W. Weimar, J. Jeekel


Br. J. Cancer (1986), 54, 337–339

### Short Communication

### Site-specific antitumour effects of 2 pyrimidinone compounds in rats

A.M.M. Eggermont[1], R.L. Marquet[2], R.W.F. De Bruin[2], W. Weimar[3] & J. Jeekel[1]

[1]*Department of Surgery;* [2]*Laboratory for Experimental Surgery and* [3]*Department of Internal Medicine I, Dijkzigt University Hospital of the Erasmus University Rotterdam, Dr Molewaterplein 40, 3015 GD Rotterdam, The Netherlands.*

Recently a series of 5-halo-6-phenyl pyrimidinones has been found to induce interferon production in several animal species (Stringfellow *et al.*, 1980) and in man (Earhart *et al.*, 1985). These compounds modulate a variety of immune responses. Most prominently they enhance the activity of natural killer (NK) cells (Taggert *et al.*, 1980) and macrophages (Li *et al.*, 1985). In the experiments reported here we evaluated the effects of two pyrimidinones that differ greatly in their ability to induce interferon (IFN) production. ABPP (2-amino-5-bromo-6-phenyl-4-pyrimidinone) induces high serum levels of IFN whereas AIPP (2-amino-5-iodo-6-phenyl-4-pyrimidinone) does not. Yet, both agents are equally active in enhancing NK cell activity (Lotzova *et al.*, 1983). In a previous communition (Marquet *et al.*, 1983) we have shown that subcutaneous growth of liposarcoma LS175 in BN rats is inhibited by IFN. This nonimmunogenic liposarcoma is of spontaneous origin and has been found to be NK resistant. In order to investigate whether the induction of IFN plays a part in the antitumour effects of ABPP and AIPP we compared the two agents in the subcutaneous and in the artificial lung metastasis model, using tumour LS175. Ten to 12-week old male BN rats were used in all experiments. The NK cell activity in peripheral blood lymphocytes (PBL) after the intraperitoneal administration of $250\,mg\,kg^{-1}$ of ABPP or AIPP was assessed by a standard 3h $^{51}Cr$ release assay as described by Ortaldo *et al.* (1977). Control animals received an equal volume of PBS i.p. The results are shown in Figure 1. A rapidly established and longlasting ±4-fold increase in NK cell cytotoxicity is seen after the administration of either agent.

The influence on macrophage activity was assessed by measuring the ingestion of latex particles by peritoneal exudate (PE) cells after a single injection of $250\,mg\,kg^{-1}$ i.p. of either pyrimidinone. Phagocytosis was rapidly 3–4 fold enhanced (within 4h) and remained elevated for 4 days (data not shown). In the s.c. tumour model 2mm cubes of tumour LS175 were implanted s.c. in the left flank of BN rats. Tumour growth was assessed on days 4, 7 and 12 by measuring the two largest perpendicular diameters of the tumour with calipers. The average diameter was taken as the measure of tumour size. The inoculation of the tumour was preceded by i.p. injections of AIPP or ABPP at a dose of $250\,mg\,kg^{-1}$ on days −3, −2 and −1. The effect of this pretreatment on tumour growth is illustrated in Figure 2. A significant inhibition of tumour growth was observed in the ABPP-treated group ($P<0.003$) whereas some, but

**Figure 1** Enhancement of NK cell activity in peripheral blood lymphocytes (PBL) 1, 3 and 9 days after a single i.p. injection of $250\,mg\,kg^{-1}$ of AIPP or ABPP. Control animals received PBS i.p. The percentage of specific lysis is shown as determined in a 3h $^{51}Cr$ release assay at an effector-to-target (Yac-1) ration of 40:1.

Correspondence: A.M.M. Eggermont
Received 4 March 1986.

© The Macmillan Press Ltd., 1986

**Figure 2** Effect of pretreatment with AIPP or ABPP on s.c. growth of tumour LS175. AIPP or ABPP were administered at a dose of 250 mg kg$^{-1}$ i.p. on days −3, −2 and −1. The difference between the mean tumour diameter in the ABPP treated group (●---●) and the control group (○---○) was statistically significant ($P<0.003$). No significant inhibition of tumour growth was observed in the AIPP treated group (the AIPP treated group (△---△). Each group consisted of 5 animals.

insignificant inhibition was seen after pretreatment with AIPP.

The same pretreatment schedule was used in the lung metastasis model. Here a single cell suspension of $10^5$ tumour cells was injected in the tail vein on day 0. The rats were sacrificed on day 14 and the number of lung colonies was counted with the naked eye after fixation of the lungs in Bouin's solution. As shown in Table I, a significantly ($P<0.02$) lower number of lung colonies was observed in the rats pretreated with ABPP or AIPP than in the control group. No difference existed between the two treated groups.

This inhibitory effect on the development of lung metastases is in agreement with the results reported by Milas *et al.* (1982) concerning immunogenic as well as non-immunogenic murine tumours.

The activity of both NK cells and macrophages at the time of i.v. tumour inoculation was strongly enhanced as a result of the pretreatment with either ABPP or AIPP. The activation of these two cells populations may well explain the results in the lung metastasis model.

Hanna *et al.* (1980) have shown that tumour cells are most sensitive to NK cell cytotoxicity when they are in a bloodborne or early lodging phase. The fact that tumour LS175 is NK resistant does not rule out an important role for activated NK cells to account for the observed lower number of lung metastases in the pyrimidone treated groups.

**Table I** Effect of pretreatment with AIPP or ABPP on the development of lung metastases after i.v. injection of $10^5$ LS175 cells

| *Treatment* | *Mean number of lung metastases* $\pm$*s.d.* | *Range* |
|---|---|---|
| PBS | 39 ± 12 | 24–56 |
| AIPP | 18 ± 7 | 10–23 |
| ABPP | 13 ± 3 | 10–16 |

Effect of administration on days −3, −2 and −1 of 250 mg kg$^{-1}$ AIPP or ABPP i.p. on the development of lung metastases, when counted on day 14 after the i.v. injection of $10^5$ cells of liposarcoma LS175. Control animals were pretreated with PBS. A significantly lower ($P<0.02$) number of metastases were observed after either AIPP or ABPP pretreatment than in the control animals. Each group consisted of 5 animals.

(i) *In vitro* NK resistant tumour cells may be less resistant *in vivo* to highly activated NK cells (Talmadge *et al.*, 1984). (ii) Very high NK cell activity can be induced in the lungs of rats by pyrimidinones (Lotzova *et al.*, 1984). (iii) Activated NK cells have been shown to produce a variety of lymphokines like IFN-gamma and IL-2 (Kasahara *et al.*, 1983).

IFN-gamma especially and a putative more rapidly working macrophage activating factor (MAF) secreted by NK cells in the rat and in man (Gomez *et al.*, 1985) are very potent activators of alveolar macrophages.

The tumour site specific setting of a high concentration of (pyrimidinone-activated) macrophages further activated by NK-cell-secreted lymphokines could well explain the observed inhibition of the development of lung metastases. The difference in tumour reponse to ABPP and AIPP in the s.c. tumour model may be related to the difference in IFN serum levels after the administration of the two agents involved. When we observe the growth curve of the tumour in the ABPP pretreated group it is noted that the tumour seems to disappear over the first few days.

This is the only antitumour effect observed since tumour growth follows a parallel course to the other experimental groups when the tumour becomes measurable. Apparently tumour suppression is only short lived and no prolonged inhibitory effect can be observed.

This could be explained by the fact that high IFN levels are induced by ABPP for a period of only 24 h (Oku *et al.*, 1984; Lotzova *et al.*, 1984). Since LS175 has been shown to be IFN sensitive and IFN can have a direct inhibitory effect on tumour growth, a high IFN level at the time of s.c. tumour inoculation may have created the ±2 day lag time in tumour growth that is observed in the animals pretreated with ABPP. Since multiple injections with ABPP induce a hyporeactive state and a fall in IFN levels (Oku *et al.*, 1984), as is observed with many biological response modifiers (Talmadge *et al.*, 1985), the antitumour effect in the s.c. model offers little prospect for an effective role for ABPP in this setting.

The anti-metastasis effect of these pyrimidinones is the more important observation, especially since the tumour used was NK resistant.

Interactions between activated NK cells and activated macrophages, both of which are present in high numbers in the lungs, may have overcome this resistance and may have brought about the effective lysis of the tumour cells by either cell population. Since the administration of pyrimidinones is virtually without toxicity or side effects (Earhart *et al.*, 1985) these agents may hold some promise in boosting anti-metastasis protective mechanisms in a peri-operative or otherwise adjuvant setting.

This study was in part supported by a grant from The Upjohn Company, Kalamazoo, Michigan, USA.

### References

EARHART, M.E., HAMILTON, R.D., HENRY, C.S. & 4 others. (1985). Phase I trial, pharmacokinetics and interferon (IFN) induction of an oral divided-dose schedule of 2-amino-5-bromo-6-phenyl-4(3H)-Pyrimidinone (ABPP) in cancer patients. *Proc. Amer. Assoc. Cancer Res.*, **26**, 159.

GOMEZ, J., POHAJDAK, B., O'NEILL, S., WILKINS, J. & GREENBERG, A.H. (1985). Activation of rat and human alveolar macrophage intracellular microbicidal activity by a preformed LGL cytokine. *J. Immunol.*, **135**, 1194.

HANNA, N. & FIDLER, J. (1980). Role of natural killer cells in the destruction of circulating tumor emboli. *J. Natl Cancer Inst.*, **65**, 801.

KASAHARA, T., DJEU, J.Y., DOUGHERTY, S.F. & OPPENHEIM, J.J. (1983). Capacity of human large granular lymphocytes (LGL) to produce multiple lymphokines: Interleukin 2, interferon, and colony stimulating factor. *J. Immunol.*, **131**, 2379.

LI, L.H., WALLACE, T.L., RICHARD, K.A. & TRACEY, D.E. (1985). Mechanism of antitumor action of pyrimidinones in the treatment of B16 melanoma and P388 leukemia. *Cancer Res.*, **45**, 532.

LOTZOVA, E., SAVARY, C.A. & STRINGFELLOW, D.A. (1983). 5-Halo-6-phenyl-pyrimidinones: New molecules with cancer therapeutic potential and interferon inducing capacity are strong inducers of murine natural killer cells. *J. Immunol.*, **130**, 965.

LOTZOVA, E., SAVARY, C.A., KAHN, A. & STRINGFELLOW, D.A. (1984). Stimulation of natural killer cells in two random-bred strains of athymic rats by interferon inducing pyrimidinone. *J. Immunol.*, **132**, 2566.

MARQUET, R.L., SCHELLEKENS, H., WESTBROEK, D.L. & JEEKEL, J. (1983). Effects of treatment with interferon and cyclophosphamide on the growth of a spontaneous liposarcoma in rats. *Int. J. Cancer*, **31**, 223.

MILAS, L., HERSH, A.M., STRINGFELLOW, D.A. & HUNTER, N. (1982). Studies on the antitumor activities of pyrimidinone-interferon inducers. I. Effect against artificial and spontaneous lung metastases of murine tumors. *J. Natl Cancer Inst.*, **68**, 139.

OKU, T., IMANISHI, J. & KISHIDA, T. (1984). Interferon counteracts pyrimidinone induced hyporeactivity and the combined treatment has antitumor effect. *Gann*, **75**, 631.

ORTALDO, J.R., BONNARD, G.D. & HERBERMAN, R.B. (1977). Cytotoxicity reactivity of human lymphocytes cultured *in vitro*. *J. Immunol.*, **119**, 1351.

STRINGFELLOW, D.A., VANDENBERG, H.C. & WEED, S.D. (1980). Interferon induction by 5-halo-6-phenyl-pyrimidinones. *J. Interferon Res.*, **1**, 1.

TAGGART, M.T., LOUGHMAN, B.E., GIBBOBS, A.J., STRINGFELLOW, D.A. (1980). Immunomodulatory effects of 2-amino-5-bromo-4-phenyl-6-pyrimidinol and its isocytosine analogs. In *Currect Chemotherapy and Infectious Diseases*, Nelson and Grassi (eds), Proc. 11th International Congress of Chemotherapy, p. 1400. American Society for Microbiology: Washington, D.C.

TALMADGE, J.E., MALUISH, A.E., COLLINS, M. & 4 others. (1984). Immunomodulation and antitumor effects of MVE-2 in mice. *J. Biol. Resp. Mod.*, **3**, 634.

TALMADGE, J.E., HERBERMAN, R.B., CHIRIGOS, M.A. & 9 others. (1985). Hyporesponsiveness to augmentation of murine natural killer cell activity in different compartments by multiple injections of various immunomodulators including recombinant interferons and interleukin 2. *J. Immunol.*, **135**, 2483.


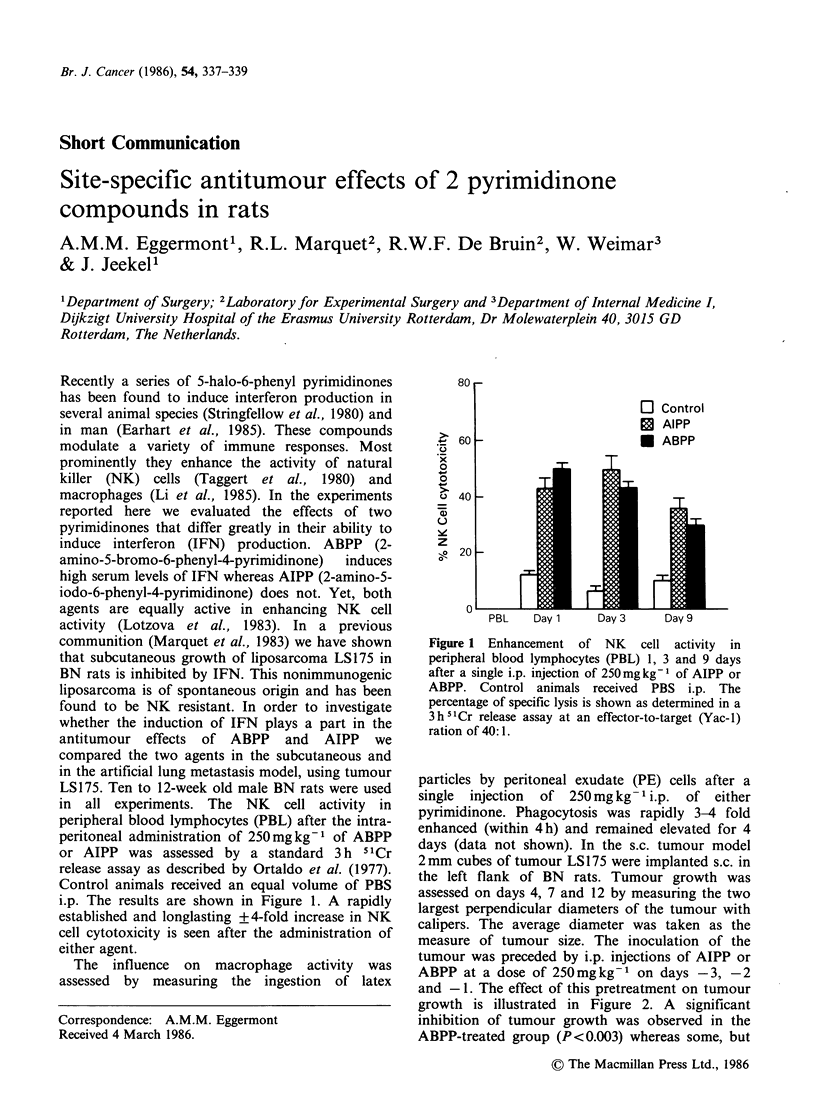

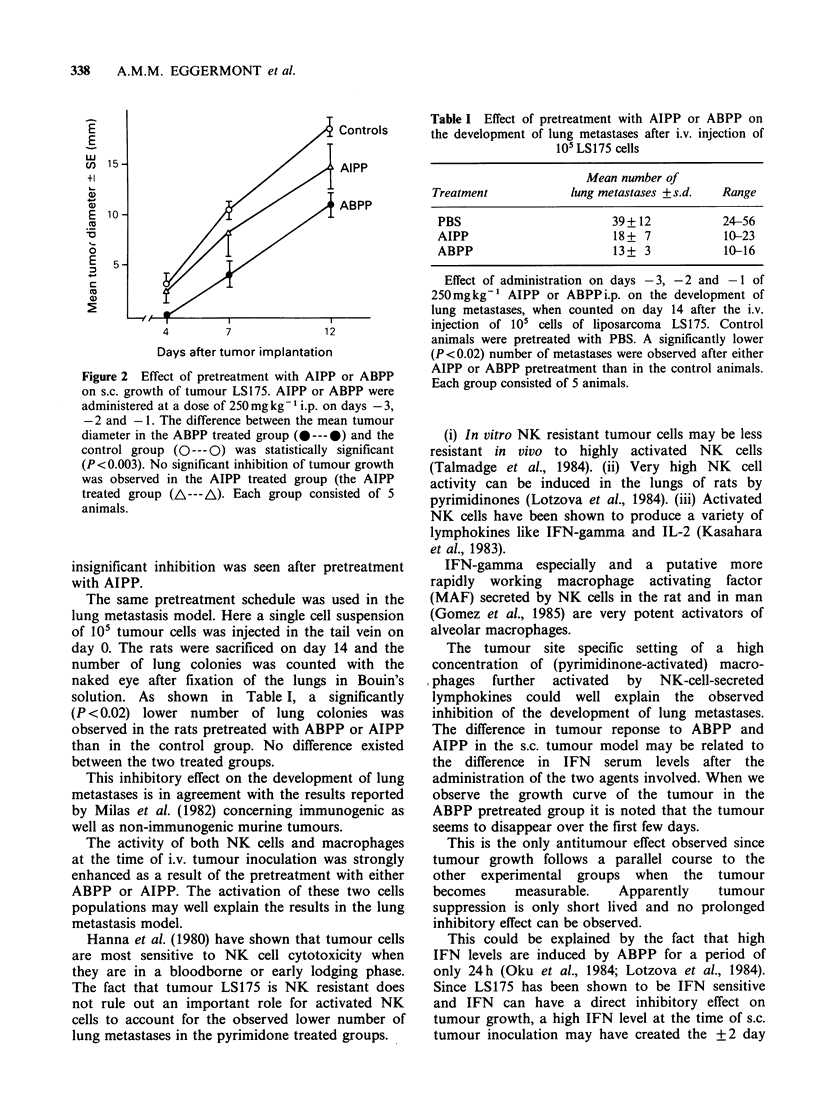

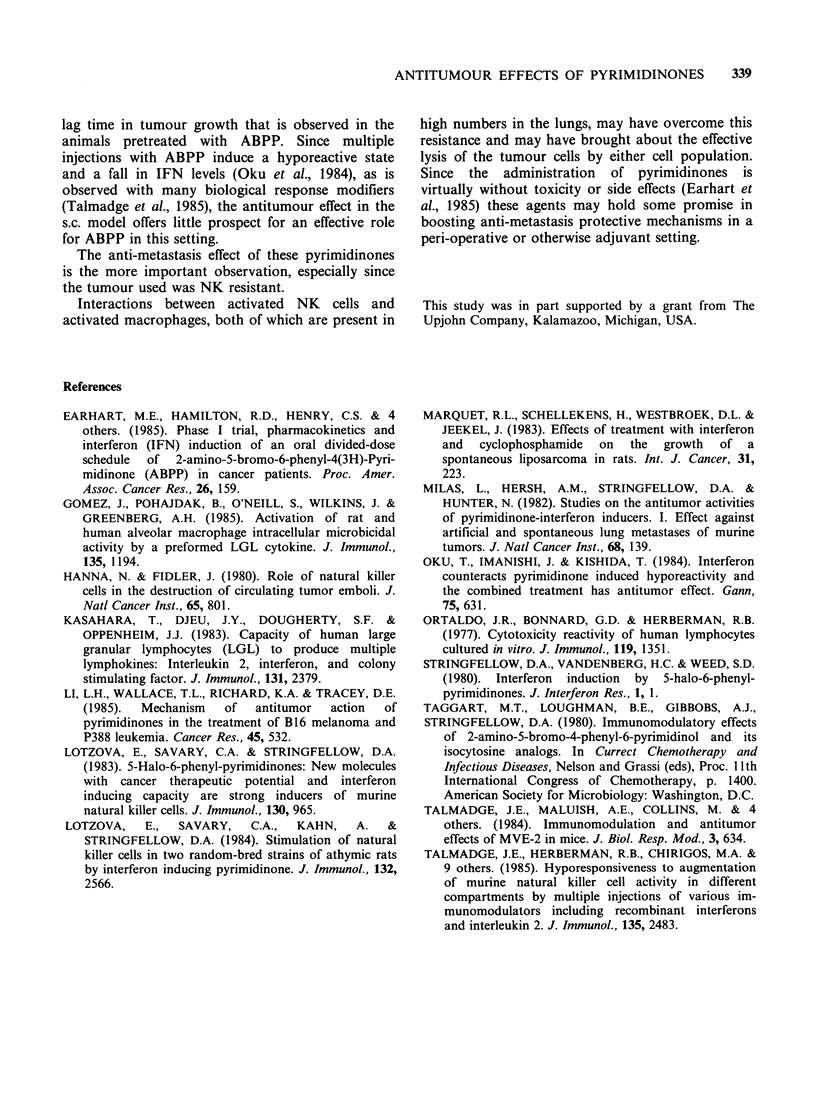


## References

[OCR_00278] Gomez J., Pohajdak B., O'Neill S., Wilkins J., Greenberg A. H. (1985). Activation of rat and human alveolar macrophage intracellular microbicidal activity by a preformed LGL cytokine.. J Immunol.

[OCR_00285] Hanna N., Fidler I. J. (1980). Role of natural killer cells in the destruction of circulating tumor emboli.. J Natl Cancer Inst.

[OCR_00290] Kasahara T., Djeu J. Y., Dougherty S. F., Oppenheim J. J. (1983). Capacity of human large granular lymphocytes (LGL) to produce multiple lymphokines: interleukin 2, interferon, and colony stimulating factor.. J Immunol.

[OCR_00297] Li L. H., Wallace T. L., Richard K. A., Tracey D. E. (1985). Mechanism of antitumor action of pyrimidinones in the treatment of B16 melanoma and P388 leukemia.. Cancer Res.

[OCR_00310] Lotzová E., Savary C. A., Khan A., Stringfellow D. A. (1984). Stimulation of natural killer cells in two random-bred strains of athymic rats by interferon-inducing pyrimidinone.. J Immunol.

[OCR_00303] Lotzová E., Savary C. A., Stringfellow D. A. (1983). 5-halo-6-phenyl pyrimidinones: new molecules with cancer therapeutic potential and interferon-inducing capacity are strong inducers of murine natural killer cells.. J Immunol.

[OCR_00317] Marquet R. L., Schellekens H., Westbroek D. L., Jeekel J. (1983). Effect of treatment with interferon and cyclophosphamide on the growth of a spontaneous liposarcoma in rats.. Int J Cancer.

[OCR_00324] Milas L., Hersh E. M., Stringfellow D. A., Hunter N. (1982). Studies on the antitumor activities of pyrimidinone-interferon inducers. I. Effect against artificial and spontaneous lung metastases of murine tumors.. J Natl Cancer Inst.

[OCR_00331] Oku T., Imanishi J., Kishida T. (1984). Interferon counteracts pyrimidinone-induced hyporeactivity and the combined treatment has antitumor effect in mice.. Gan.

[OCR_00337] Ortaldo J. R., Bonnard G. D., Herberman R. B. (1977). Cytotoxic reactivity of human lymphocytes cultured in vitro.. J Immunol.

[OCR_00342] Stringfellow D. A., Vanderberg H. C., Weed S. D. (1980). Interferon induction by 5-halo-6-phenyl pyrimidinones.. J Interferon Res.

[OCR_00360] Talmadge J. E., Herberman R. B., Chirigos M. A., Maluish A. E., Schneider M. A., Adams J. S., Philips H., Thurman G. B., Varesio L., Long C. (1985). Hyporesponsiveness to augmentation of murine natural killer cell activity in different anatomical compartments by multiple injections of various immunomodulators including recombinant interferons and interleukin 2.. J Immunol.

[OCR_00355] Talmadge J. E., Maluish A. E., Collins M., Schneider M., Herberman R. B., Oldham R. K., Wiltrout R. H. (1984). Immunomodulation and antitumor effects of MVE-2 in mice.. J Biol Response Mod.

